# Transforming students’ green behavior through environmental education: the impact of institutional practices and policies

**DOI:** 10.3389/fpsyg.2024.1499781

**Published:** 2025-01-15

**Authors:** Cai Chen, Pomi Shahbaz, Shamsheer ul Haq

**Affiliations:** ^1^Sichuan Digital Economy Industry Development Research Institute, Xi’an Jiaotong University, Chengdu, China; ^2^Department of Economics, Division of Management and Administrative Science, University of Education, Lahore, Pakistan

**Keywords:** environmental sustainability, environmental education, higher education institutions, pro-environmental behavior, responsible consumption

## Abstract

Environmental education is crucial for achieving ecological sustainability goals and transforming human behavior to promote responsible consumption. Higher education institutions play a fundamental role in transforming societies aimed at a more sustainable future through the dissemination of environmental education to millions of young people worldwide. Therefore, this study aimed to measure the role of environmental education in transforming students’ green behavior (SGB), along with other higher education institutional factors such as green campus initiatives (GCI), institutional ecosystem (IEC), institutional sustainability system (ISS), and institutional support system (ISP) through students’ green intentions (SGI). The study data were collected from 480 Chinese students enrolled in the four cities with highest number of higher education institutions through face-to-face cross-sectional survey. The collected data were analyzed through partial least squares structural equation modeling. The findings indicate a significant and positive impact of GCI, IEC, ISS, and ISP on SGI, which further positively affects the SGB. This implies that green initiatives at campus, green ecosystems, sustainable and environmentally oriented policies, and support systems of educational institutions greatly contribute to the development of students’ green intentions (SGI), which further turn into green habits fostering their green behavior. Moreover, environmental education also played a significant moderating role between SGI and SGB. The provision of support systems, organizing hands-on workshops and seminars, providing sustainable food items at cafeterias, and short campus visits focusing on campus green practices may inspire students to adopt green practices in their daily routines.

## Introduction

The environment is worsening at a rapid pace across the globe owing to a web of social-ecological challenges, including, but not limited to, climate change (Barnosky and Hadly, 2016). Researchers working on these deteriorating global ecological conditions have stressed the importance of resilient ecosystems for the survival of living organisms (UN Environment, 2019). Many scientists recommend that sustainable consumption and production behavior be the key to protecting and reducing global environmental hazards (Mastr’angelo et al., 2019). Green behavior is critical to achieving environmental sustainability, and environmental education (EE) plays a vital role in transforming individuals’ green consumption behavior.

Green behavior is not merely an outcome of individuals’ interpersonal characteristics, but also arises from social settings. Environmentally friendly social settings have become important determinants of green behavior, resulting in green consumption (Trong et al., 2023). Individuals’ daily activities affect environmental sustainability. For this reason, green behaviors can significantly shrink greenhouse carbon footprints (Dubois et al., 2019) because several individual-level actions would result in noteworthy positive ecological impacts (Christie et al., 2021). In this sense, EE can act as a catalyst to initiate green behavior. EE provides synergistic spaces for environmental researchers, policymakers, society members, and other relevant ecosystem stakeholders to converge. Synergistic space to interpret and apply the results of environmental research in real social, political, and economic contexts is required to solve environmental sustainability challenges, instead of merely sharing these findings with policymakers (Knight et al., 2019).

EE is an approach that literate individuals about their ecological responsibilities and addresses environmental sustainability issues (Wheaton et al., 2018). It promotes environmental sustainability through the transformation of human behavior and fostering environmental values (Mastr’angelo et al., 2019). Moreover, EE is not age restricted and remains relevant throughout an individual’s life (Leal Filho et al., 2018a, 2018b). Lifelong impact is essential for environmental challenges, requiring sustained thinking, involvement, and decision-making not only as individuals but also within collective actions. It also emphasizes outcomes at different levels, such as individual, community, and ecosystem. Based on prior studies, considering individuals’ behavioral complexity, EE has diverted from its traditional linear path from conservational attitudes to awareness of action, now stressing an active, multifaceted ecosystem of associations that affect individual behavior (Marcinkowski and Reid, 2019).

Keeping in view the interdisciplinary nature of environmental studies, ecological scientists now include principles from diverse disciplines, including but not limited to human psychology, education, sociology, economics, and the natural sciences (Jacobson et al., 2015). The diverse nature of perceptions and theoretical frames leads scientists to foresee it as a real strategy in the field of environmental sustainability. These strategies include having direct experience, common environmental values, building a relationship with the local environment, developing and improving action-based skills, and having opportunities to use those skills for important problems (Monroe et al., 2017).

Environmental stakeholders (researchers, policymakers, funders, and other parties) continuously call for evidence of the tangible role of EE in achieving ecological sustainability. Scientists constantly call for exploration of the relationship between EE and ecological outcomes (Coulombe et al., 2020). Likewise, environmental stakeholders want to understand the processes that underpin environmental stewardship even though variability and complexity make it difficult to examine these complex relationships (Johnson et al., 2012. Ardoin et al., 2018). Reflecting on the concept of EE deepen ecological practices and behavior change for sustainable consumption (Toomey et al., 2017). EE has desired goals in the short, intermediary, and the long term. Thus, outcomes of engaging in these objectives define success of EE programs (Ardoin et al., 2018). The rationale for examining the joint between EE programs and sustainable outcomes could be potentially further restricted because of extending scope (Ardoin et al., 2013). EE programs also involve several actors who work constructively to achieve optimum results in a rather complicated system.

Hence, EE has a significant role in facilitating the attainment of environmental sustainability objectives as well as influencing consumer behavior. Numerous studies have been carried out to establish the relationships between EE and individuals’ green behavior (Varela-Candamio et al., 2018; Woo, 2021; Salazar et al., 2024; Mullenbach and Green, 2018; Van De Wetering et al., 2022; Ardoin et al., 2020). One of the major limitations in the green behavior literature is determination EE impact on a specific consumption behavior like recycling, bus use, or green product purchase. Second, there is a lack of research works on students’ green behavior (SGB) and EE. Third, we failed to locate any study that has endeavored to examine the role of HEIs on students’ daily green behavior (SGB). Therefore, this study examines the role of HEIs environment on sustainable consumption of students. Specifically, this study aims at the analysis of the relationship between green campus initiatives (GCI) institutional ecosystem (IES) institutional sustainability policy (ISP) institutional support system (ISS) and students’ green intentions (SGI) The second research question of the present study was as follows: What is the link between SGI and SGB? Additionally, this study tests the moderation effect of EE on the relationship between SGI and SGB.

After confirming the internal reliability and validity of constructs, the partial least square structural equation model reveals the significantly positive impact of GCI, IEC, ISS, and ISP. It signifies that the campus engaged with green initiatives, greatly focus on green ecosystem, with strong support system and sustainable policies can play crucial role in developing the SGI of the students. These intentions foster their adoption of green habits leading to green behavior. Moreover, the significant positive moderating impact of EE indicates that the students with EE can act as strong moderator of the effect of SGI on SGB.

This study adds to the literature in three ways. This is the first study that considers the daily green behavior of students and its influencing factors instead of investigating a specific purchasing green behavior. This would assist HEIs’ administration and authorities in understanding the motivation behind young consumers’ daily green intentions. Second, relating EE alongside other HEIs catalysts with SGB is a noble idea. Third, this study considers a unique set of variables, such as GCI, IES, ISS, ISP, SGI, EE, and SGB, to explore their complex relationships. Thus, this study will assist in determining the most important factors in the university environment that motivate GE intentions, which may then be utilized to formulate policies to encourage SGB. This research can also assist in achieving the United Nations’ Sustainable Development Goals (SDGs), such as SDG 12 (Responsible Consumption and Production) and SDG 13 (Climate Action).

## Literature review and hypotheses development

HEIs are adopting policies and practices to reduce their carbon footprint and foster environmental sustainability (Ribeiro et al., 2021; Leal et al., 2024). These sustainable measures include recycling, waste reduction, energy and water conservation, and tree plantation (Velenturf and Purnell, 2021). Students are the focal point of such policies and practices, as their green intentions significantly impact the ecological sustainability campaigns of HEIs (Frizon et al., 2024).

The challenges have seen HEIs take measures to engage in the efficient use of renewable energy, green transport, and recycles intuitionally (Leal Filho et al., 2023). Students are the biggest beneficiaries of HEIs’ environmental sustainability plans because they are the main consumers of the institutions’ resources hence can support the sustainability process by switching off all the lights in any unoccupied room, using energy efficient appliances and avoiding wastage. HEIs promote the use of materials and recycling, and avoid the use of local resources, water containers among others (Coy et al., 2013). Likewise, HEIs encourage the students to cycle, use electric cars, public transport and sharing of cars. Some of the policies promulgated in the campuses encourages students to commute on foot or on bicycles with an aim of minimizing the carbon emissions. Sisriany and Fatimah (2017) have also mentioned that GCI minimizes carbon footprint of HEIs. They also embrace water-saving measures through installation of water-saving technologies including low-flow toilets technological measures in addition to water-saving campaigns. Likewise, the campaigns in the community garden and the campus plantation help promote students’ environmentalism. Therefore, the findings indicate that students with the access to eco-friendly infrastructure in their HEIs are likely to actualize their objectives in line with sustainability goals of the organization. Therefore, we assume.

H1: GCI positively affects SGI.

This means that the HEIs ecosystem has a very crucial role to play in influencing the SGI and thereby the achievement of SDGs (Ketlhoilwe et al., 2020). In another study, Chen et al. (2024) posited that through possessing sustainable infrastructure including; buildings, renewable energy, environmentally friendly transport systems and recycling facilities HEIs demonstrate a clear commitment towards environmental sustainability. The sustainable policies and practices of HEI also influence SGI to act in the same manner in their day to day lives, (Almarshad, 2017). For instance, the familiarization with the renewable efficient energy systems used in HEIs also influences the students’ perishable intentions to adopt the energy systems at home. If there is provision for environment conservation related structures within the HEIs, the students are likely to have their intentions blend well with their organizations’ sustainable development plans (Sousa et al., 2022).

The sustainable practices of HEIs are an absolute necessity for shaping SGI. Environmental goals are becoming a part of the curriculum, with either environmental courses or sustainability as a major. HEIs affect students’ intentions towards environmental conservation (Elegbede et al., 2023). Coy et al. (2013) noted that when sustainability is incorporated into students’ curriculum, green intention intensifies because students acquire knowledge and skills in managing environmental sustainability issues (Weiss et al., 2021). In addition, a variety of institutional policies, including the recycling policy across campuses, the prohibition of single-use plastics, the rewarding of sustainable means of transport, and others engender green practices. These guidelines are usually followed by students so that there is a positive intention towards eco-friendly practices on campus, as well as off-campus activities (Freeman-Green et al., 2023). Thus, the green intentions are also tied to the campus culture and social norms of the students. The level of engagement in environmental activism and the perceived actions of peers can affect the level of a student’s commitment toward sustainable development (Finnerty et al., 2024). This study hypothesizes.

H2: IES significantly affects SGI.

Still, the extent to which service operations have been realigned with international environmental standards and legislation has been seen to some extent in organizations’ strategic and policy documentation, where organizations have begun to integrate environmental goals (Chung, 2020). Self-organized policies, such as institutional policies and green infrastructure, directly impact students’ green intentions through support and direction-offered HEIs. Such policies can include waste disposal, energy conservation, or environmentally friendly transport policies that determine environmental consciousness among students (Park and Park, 2024). When HEIs set up clearly defined sustainability policies, their implementation allowed students to maintain and sustain environmental objectives. Hence, they adopt green lifestyles while in HEIs, and even in their future endeavors (Biancardi et al., 2023).

There is one major area where HEI policies affect students: waste management. Campuses use different sustainable practices, such as implementing a recycling bin program or a composting program, and implementing policies that prohibit the use of plastic utensils or disposable containers (Diestro, 2022). These policies will assist in demystifying sustainable practices among students. It is easier for them to adopt sustainable practices as their way of life. Researchers have also suggested that when universities embrace the culture of reducing waste, students will have better green intentions. For instance, a cross-sectional survey conducted by van Geffen et al. (2020) to determine the environmental consciousness of students in the context of campus recycling and waste reduction showed that students who participated in campus recycling and waste reduction programs displayed high levels of environmental consciousness. HEIs’ commitment to sustainability ensures that students are accountable for their environmental footprints, both within and outside the campus. Therefore, energy efficiency measures have a direct relationship with students’ green attitudes. Munaro and John (2024) discussed renewable energy systems on campuses and found that such investments increase student support for clean energy. When students witness these policies being implemented, they will promote such behaviors, including switching off appliances not in use or even pushing for the promotion of renewable energy sources in their future workplaces.

Likewise, environmentally sound transport policies make students consider how they can change the ways they get to and from the campus. According to Zhou’s (2016) study on green transport at universities, student transport behavior revealed that universities contribute to environmentally friendly transport practices. Such policies are in line with students’ concerns about the environmental consequences of traditional means of transport and the push for the adoption of environmentally friendly means of transport. In summary, one may conclude that evidence of SGI is closely related to HEI policies. According to the person-organization (P-O) fit theory, institutional factors (manifested by HEI green policies and practices) may have a significant influence on SGI (Renwick et al., 2016). We also hypothesize.

H3: ISP has a significant relationship with SGI.

H4: ISS is positively associated with the SGI.

The association between green intentions and green behavior is a significant topic for environmental researchers (Wang, 2024). Parker et al. (2023) found a positive correlation between green intentions and green behaviors of individuals. Similarly, Chwialkowska et al. (2024) found a significant relationship between green intentions and subsequent green behavior. Joshi et al. (2021) conducted a study on recycling behavior and noted a significant positive impact of green intentions on green behavior. Yu et al. (2019) state that strong green intentions lead to environmental sustainability. Responsible consumption behavior is essential for achieving green behavior among individuals (Kebede et al., 2023). Individuals with green intentions are more likely to adopt green behavior in their real lives to protect and foster environmental sustainability. We also hypothesize:

H5: SGI positively affects the SGB.

EE is a vehicle that changes people’s behavior and saves the natural environment by sharing information and concern about the important topic of sustainability. It also enhances the processes required in the fixing of the environmental complications. Zsóka et al. (2013) confirmed that EE has positive and significant effect on students’ green purchasing behavior. Elsewhere Al-Nuaimi and Al-Ghamdi (2022) posited that EE is important in defense and enhancement of the environment through provision of information to the wider community. EE helps people to evaluate the benefit or cost of any given action. This makes it possible for one to have corrective action plans in efforts to avoid negative effects and increase positive ones in promoting green practices. Thus, we hypothesize that.

H6: EE moderates the relationship between SGI and SGB.

**Figure 1:**
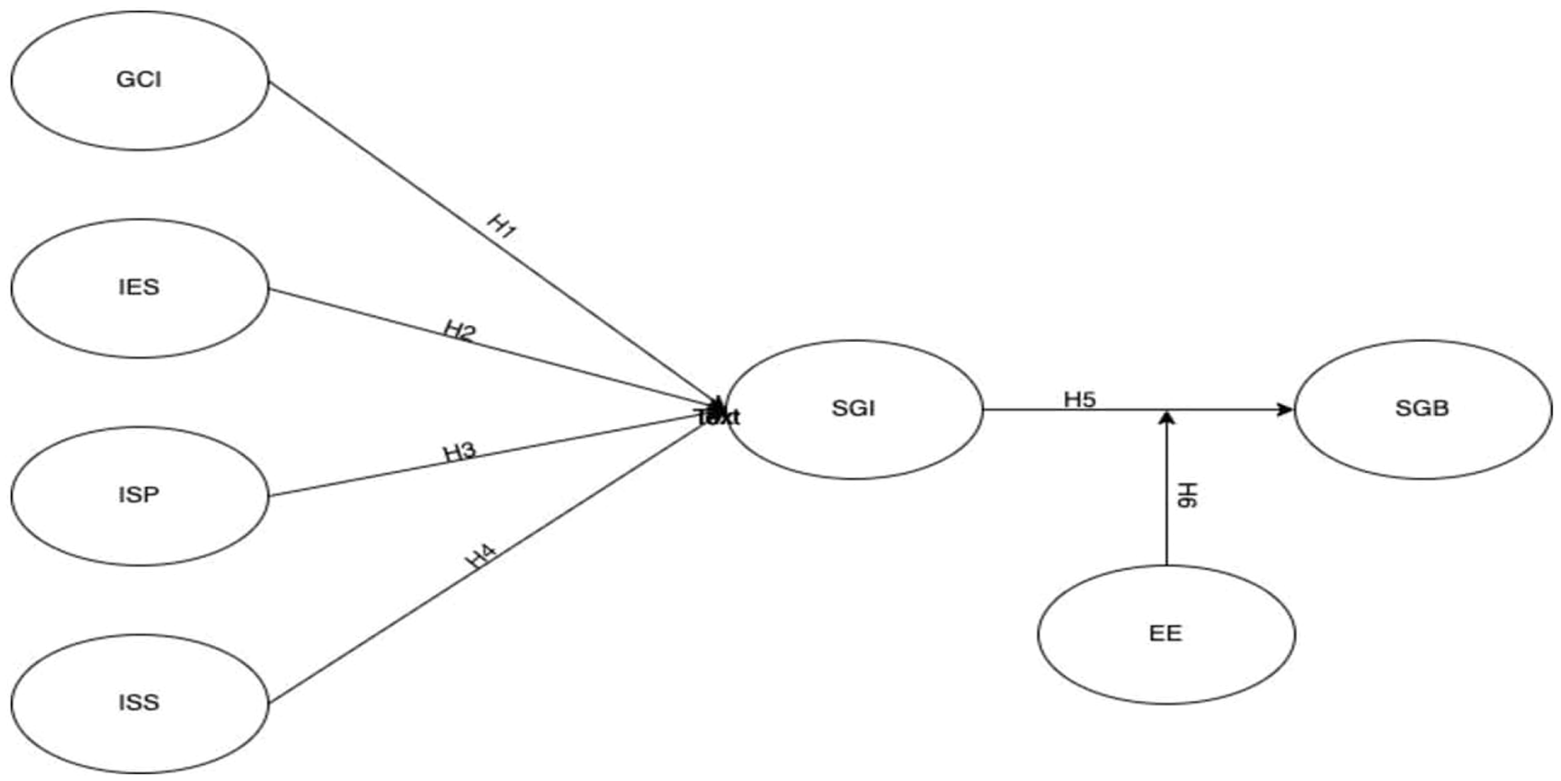
Hypothesis and constructs. SGB, students’ green behavior; SGI, Students’ green intentions; IES, Institutional ecosystem; ISP, Institutional sustainability policies; ISS, Institutional support system.

Figure 1 shows the relationships hypothesized among different variables in this study.

## Materials and methods

### Study design and survey instrument

To attain the environmental sustainability goals in each field there must be real inputs irrespective of the field and sector (Han et al., 2019). Thus, it seems rational to examine the determinants of students’ environmental behavior in HEI contexts to enhance SGB for sustainability. Scholars have found that HEIs have profound relations with the ecological systems and these institutions can contribute greatly to minimizing the degradation of the environment (Coulombe et al., 2020). Consequently, the HEIs possess a great potential in the preservation or ecological systems by means of the institutional approaches and measures (Ruiz-Mallén and Heras, 2020). HEIs paly its crucial role in achieving the environmental sustainability by providing the critical information about the climate change, and can raise the awareness among students about causes of climate change and its mitigation strategies (Esakkimuthu and Banupriya, 2023). Similarly, HEIs can provide a platform at which students can practice the eco-friendly practices such as energy saving and recycling (Kitheka, 2024). In addition, HEIs are learning institutions for millions of youths who will be in a position to protect natural resources globally within the next few years and contribute towards the realization of environmental sustainability. Hence, identification of the variables influencing SGB in context to HEI setting is crucial to fulfil the goals of sustainable consumption and production.

The population of this study consisted of 400 million young minds enrolled in more than 2900 HEIs in China (China Daily, 2023). This youth represents a considerable proportion of consumers in China, and they will be future policymakers in different sectors, making them an important segment of present and future environmental policies. Students enrolled in the top four cities with the highest number of HEIs (Beijing, Wuhan, Guangzhou, and Zhengzhou) were selected to represent the student community of China (China Daily, 2022). This study used a quantitative research design through a cross-sectional survey to collect data from 480 students from 16 selected 16 HEIs in these cities. The survey instrument was prepared by taking insights from relevant literature and finalized after incorporating the few changes suggested by the research team after pre-testing survey. The pre-testing was conducted with 20 students, and these surveys were not included into the final analysis. The teams distributed survey instrument to the students in the selected universities. Data were collected using paper-and-pencil questionnaires, and all participants were approached in person by asking for their participation.

The survey instrument was validated in two steps before starting the final survey. Content validation was performed by consensus of experts and then by conducting a pretest. Unclear and irrelevant information was removed during the validation. The survey instrument was prepared in Chinese language for better understandings of study participants. The survey was divided into two sections. The first section consisted of questions related to students’ sociodemographic characteristics. The average age of the students was 24.35 years, and most of them were living at hostels. Moreover, 68.49% students reported that they have ever taken a course regarding the environment. The second section consisted of questions aimed at measuring the factors affecting SGB at HEIs on a 5-point Likert Scale (1 for strongly disagree, 2 for disagree, 3 for neutral, 4 for agree and 5 for strongly agree).

### Statistical technique

The study used descriptive statistics and partially least square structural equation modeling (PLS-SEM) to analyze the data from students and explore the associations between the different constructs. This model can measure both direct and indirect relationships among a number of latent variables together. PLS-SEM is made up of inner and outer models. PLS-SEM does not suppose about the variance of the data (Vinzi et al., 2010). Therefore, this study preferred to utilize PLS-SEM for analysis in this study.

## Results

Table 1 provides important information about students’ perceptions of their green behavior and intention, along with various underlying constructs. The data entailed information about the individual items used to measure the underlying construct.

**Table 1:** Analyzing individual items and convergent validity of measurement model.

Constructs	Mode	Mean	S.D.	Factor loadings	Cronbach alpha	CR	AVE
Students’ green behavior (SGB)		4.16	1.17		0.856	0.980	0.777
SGB1	4	3.85	1.07	0.936			
SGB2	5	4.83	1.17	0.918			
SGB3	4	3.79	1.29	0.893			
SGB4	4	3.65	1.27	0.878			
SGB5	3	2.88	1.09	0.854			
SGB6	4	3.89	1.03	0.844			
SGB7	4	3.87	1.18	0.837			
SGB8	5	4.93	1.18	0.825			
SGB9	4	3.69	1.33	0.819			
SGB10	5	4.87	1.31	0.806			
SGB11	5	4.94	1.11	0.796			
SGB12	4	3.59	1.06	0.787			
SGB13	5	4.75	1.15	0.776			
SGB14	5	4.77	1.19	0.767			
Students’ green intentions (SGI)		4.23	1.19		0.891	0.967	0.746
SGI1	5	4.86	1.26	0.952			
SGI2	4	3.69	1.19	0.949			
SGI3	4	3.75	1.31	0.931			
SGI4	4	3.89	1.07	0.903			
SGI5	4	3.92	1.05	0.884			
SGI6	4	3.71	1.11	0.859			
SGI7	5	4.72	1.21	0.844			
SGI8	4	3.85	1.31	0.831			
SGI9	5	4.92	1.25	0.812			
SGI10	5	4.95	1.16	0.804			
Green campus initiatives (GCI)		4.67	1.13		0.809	0.944	0.771
GCI1	5	4.96	1.15	0.879			
GCI2	5	4.77	1.04	0.856			
GCI3	5	4.82	1.06	0.833			
GCI4	4	3.91	1.19	0.829			
GCI5	5	4.89	1.22	0.806			
Institutional ecosystem (IES)		4.31	1.26		0.821	0.938	0.791
IES1	5	4.68	1.29	0.855			
IES2	4	3.90	1.32	0.849			
IES3	4	3.75	1.16	0.829			
IES4	5	4.91	1.27	0.811			
Institutional sustainability policies (ISP)		4.16	1.21		0.811	0.925	0.673
ISP1	4	3.83	1.21	0.916			
ISP2	4	3.9	1.13	0.907			
ISP3	5	4.85	1.04	0.849			
ISP4	4	3.67	1.33	0.822			
ISP5	5	4.79	1.41	0.816			
ISP6	4	3.94	1.11	0.801			
Institutional support system (ISS)		4.25	1.04		0.856	0.920	0.741
ISS1	5	4.66	1.11	0.904			
ISS2	4	3.72	1.08	0.868			
ISS3	4	3.85	1.19	0.838			
ISS4	5	4.77	1.13	0.813			

S.D., standard deviation; AVE, average variance extracted; CR, composite reliability.

### Students’ green behavior

The average score of SGB equal to 4.16 indicates that students are consciously engage with the environmentally friendly action frequently leading to foster SGB. The standard deviation of 1.17 signifies that while many students frequently exercise SGB, there are some differences in how actively they adopt green actions. The findings regarding individual items reveal that many students prefer to repair and reuse broken items instead of buying them immediately (SGB11 = 4.94), and are also diligent in lowering their food waste (SGB8 = 4.93). Similarly, they also had a strong preference for locally produced or organic foods to decrease their carbon footprint (SGB10 = 4.87). Moreover, they eagerly conserved energy by turning off lights and appliances when not in use (SGB2 = 4.83). Most of the students indicates that they would like to participate actively in the environmental protection activities (SGB14 = 4.77), and they also prefer to use the energy efficient appliance and light bulbs in order to lower the energy use (SGB13 = 4.75). Additionally, the students also indicated a high level of agreement on preparing reusable shopping bags to improve the use of plastic bags (SGB6 = 3.89), frequently recycling recyclable materials such as paper and plastic (SGB1 = 3.85), paper-saving habits by avoiding unnecessary printing (SGB7 = 3.87), saving water while brushing teeth, and taking showers by closing running taps (SGB3 = 3.79). On the other hand, choosing a reusable water bottle over a single-use water bottle (SGB5 = 2.88) and the disposal of hazardous materials such as batteries and electronics through designated recycling programs (SGB12 = 3.59) were less frequently adopted by the students.

### Students green intentions

The average SGI (=4.23) indicates that students have strong green intentions and are more committed to eco-friendly behavior. This implies that students generally intend to adopt green practices in their daily routine activities. The standard deviation of 1.19 indicates that most of the students signify a similar level of intention toward living greener, with slight differences. Considering their specific intentions, they indicated a strong commitment to saving water and lowering waste (SGI10 = 4.95) and using the energy efficient appliances at home (SGI9 = 4.95). This indicates their proactive approach toward lowering environmental implications in their daily lives. They depicted their intention to adopt more environmentally friendly habits in general, such as waste reduction and energy conservation (SGI1 = 4.85), and were highly inclined to support sustainability initiatives of campus and community (SGI7 = 4.72). Moreover, students are more likely to educate themselves on environmental issues to make more informed and green choices (SGI5 = 3.92), prefer public transport for lowering carbon footprints (SGI4 = 3.89), and are more inclined to adopt waste reduction and recycling activities in daily routine activities (SGI8 = 3.85). In certain areas, students depicted a moderate level of intention. For example, they were not more anxious about choosing green products while purchasing (SGI2 = 3.69).

### Green campus initiatives

The findings about GCI show that students have a strong engagement with the initiatives taken by campus. Moreover, it depicts the positive effects of these green initiatives on students. The average score of 4.67 indicates that students generally have a strongly high level of agreement with campus initiatives and their influence on them. A mode equal to 5 for GCI1 indicates that students frequently use recycling bins on campus. Similarly, a high average GCI1 score (=4.96) with a low standard deviation (=1.15) reflects strong engagement with recycling. GCI2 is about the influence of an energy-saving program on the daily habits of students, and most students have shown the highest level of consensus over the impact of programs on their daily routine actions. The students also indicated the highest level of agreement with GCI3 (=5), which reflects that they feel that the university’s facilities strongly support sustainable living. A mode equal to 4 and average scores equal to 3.91 (GCI4) signifies that many students are involved in initiatives such as waste reduction and water saving on campus, but show slightly less overall engagement as compared to the other GCIs. Moreover, the students again indicate the highest level of agreement over the impact of the university’s green initiatives on students to adopt green practices outside the university. A mode of 5 and a high average score (=4.89) confirm that university initiatives have a strong impact on students’ daily routine actions beyond the campus.

### Institutional ecosystem

The institutional environment is also important in the development of students’ green intentions, leading to green behavior. The average of 4.31 IES reflects that most students have shown high consensus over the supportive environment of campus that promotes sustainability. Considering the individual items, IES1 with a mode of 5 indicated that most students indicated the highest level of agreement with the supportive campus environment that encourages sustainable practices. The mode of IES2 = 4 reflects that student agreed with the provision of resources and infrastructure to support green behavior, but the average was 3.90, with a standard deviation of 1.32, indicating that students generally feel that sufficient resources are provided by the university, but there is still a need for improvement. Similarly, most students scored 4 on the physical layout and design of the campus promoting green practices (IES3). The lowest average of IES3 (=3.75) as compared to all other IES items indicates that the design and layout of the campus is not strongly perceived as promoting sustainability by the students. The highest mode (=5) and average (=4.91) of IES4 reflect that student generally strongly perceived that their campus culture and daily operations highly integrated sustainability.

### Institutional sustainability policies

Considering the ISP, an average score of 4.16 indicates that students generally perceived university policies focusing on sustainability. The average of ISP1 (=3.83) indicates that the students are generally aware of the university’s long-term sustainability goals, while a standard deviation of 1.27 indicates there is still a need for improvement in raising awareness among the students. Similarly, students also show that university policies encourage them to follow green practices with a mode of 4 and an average of 3.90, with a standard deviation of 1.13, indicating that most students feel encouraged to adopt green practices, although some of them may perceive that the policies could be more motivating. ISP3 reflects that students show the highest level of agreement with the direct link between university policies and positive environmental changes on campus. This implies that students feel that the university’s policies have a strong favorable impact on the campus environment. The mode of ISP4 is equal to 4, and average scores equal to 3.67 with a slightly high standard deviation (=1.33), reflecting that students believe the university policies are effective in lowering the ecological footprints, but there is a variation in responses. The response to ISP5 indicates that students are strongly inspired by university sustainability policies to become environmentally responsible according to a mode equal to five and an average score of 4.79. The highest deviation 1.41 indicates a significant variation in responses among the students. The average ISP6 score is 3.94, indicating that students perceive that university communication is clear and motivating regarding its sustainability goals.

### Institutional support system

The ISS indicates the support system at the university, which provides important information about how students perceive this system to be supportive. The overall average ISS of 4.25 indicates students strongly perceive that the university system supports sustainability at campus. Students strongly agree that their institution provides opportunities to learn about sustainability (ISS1 = 4.66) and supports the sustainability projects of students (ISS4 = 4.77). On the other hand, students indicated a low perception level of the clarity of policies promoting sustainability programs (ISS2 = 3.72), and that the institution encourages their students to participate in sustainability programs (ISS3 = 3.85) as compared to other ISS items.

### Validity assessment of measurement model

The current study examines two types of validity of measurement: convergent validity (CV) and discriminant validity (DV). Factor loadings (FL) indicate how strongly individual items relate to their underlying constructs. This reflects the magnitude of the relationship between the items and the underlying construct. A high FL indicates a strong relationship between an item and its construct. The threshold value for FL is 0.70 (Cheung and Rensvold, 2002; Su et al., 2023), and items having FL below 0.70 must be removed to enhance the CV of the model (Ma et al., 2023). The results indicates that the items under each construct secure FL values greater than 0.70, which confirms the CV of the model.

Cronbach alphas was used to establish the internal reliability of these constructs. It determines the amount of homogeneity between items in relation to an assumed construct. Often such a result proves the effectiveness of the scale developed to assess an affective construct (Pan et al., 2024). The Cronbach alpha’s score ranges between 0 and 1. A closer value to 1 depicts that there was higher interrelatedness of the items in a construct showing sign of internal consistency and reliability. It is evident from the results that the alpha score for each of the construct is more than 0.80. Hence internal consistency and reliability is maintained which leads to higher CV of the model.

Composite reliability (CR) was also measured to provide more effective measurement of internal reliability as compare to FL. Zhang et al. (2024) states that the value of CR should not be less than 0.60 for construct validity. Moreover, CR greater than 0.70 signifies the sufficiency of the model (Pan et al., 2024). While its value greater than 0.80 is highly recommended to confirm the adequacy of model (Chin, 2009). The findings related to CR confirm the internal reliability and adequacy of model leading allowing researchers to further analysis. Average variance extracted (AVE) explains that how much variance in observed variables of underlying construct. The findings related AVE values greater than 0.50 showed that the measured constructs substantially explained the variance in observed variables.

Table 2 presents the findings of Fornell-Larcker Criterion (FLC) and Heterotrait-Monotrait Ratio (HMR) to confirm the DV of the measurement model. DV indicates how a construct is distinct from all other constructs in a model. FLC indicates the correlation scores among all constructs, and these scores are compared with the square root of the AVE of a specific construct. If the correlation score of a construct with all other constructs is lower than the square root of the AVE of that specific construct, then it confirms DV (Rahman et al., 2021). The results indicates that the diagonal elements of the FLC correlation matrix are greater than the correlation scores, implying that the specific construct signifies greater variability with its own measurement items than with other measures. Similarly, HMR also confirms DV, as its values below 0.90 indicates the highest DV of the model (Rouf and Akhtaruddin, 2018).

**Table 2:** Discriminant validity.

	SGB	SGI	GCI	IES	ISP	ISS
Fornell-Larcker criterion						
SGB	0.881					
SGI	0.421	0.864				
GCI	0.183	0.391	0.878			
IES	0.204	0.253	0.473	0.889		
ISP	0.297	0.295	0.473	0.311	0.820	
ISS	0.105	0.344	0.284	0.284	0.402	0.861
Heterotrait-Monotrait ratio						
SGB						
SGI	0.384					
GCI	0.184	0.374				
IES	0.311	0.229	0.277			
ISP	0.174	0.206	0.133	0.401		
ISS	0.207	0.343	0.284	0.302	0.223	

SGB, Students’ green behavior; SGI, Students’ green intentions; IES, Institutional ecosystem; ISP, Institutional sustainability policies; ISS, Institutional support system.

### Goodness of fit of structural model

Table 3 confirms the goodness of fit of the SEM model, as all the parameters encompass the threshold limits. For example, χ2/df is equal to 2.5, which remains below the cut-off value (<3.0). Similarly, for a well-fitted SEM, the root mean square error of approximation (RMSEA) is lower than 0.08, which is equal to 0.057. Similarly, all other parameters had values lower than the threshold limits. These findings support the need for further research.

**Table 3:** Model goodness of fit estimation.

Fitness tests	Critical values	Computed values
Χ2/df	<3.0	2.5
GFI	>0.90	0.931
AGFI		0.92
CFI		0.915
NFI		0.923
RMSEA	<0.08	0.057

GFI, Goodness of fit index; AGFI, Adjusted goodness of fit index; CFI, Comparative fit index; NFI; Normed fit index; RMSEA, Root mean square error of approximation.

### Path analysis

Table 4 shows the findings of SEM model. The R^2^ in the table is the parameter of explained variance, which indicates the predictive accuracy of SEM. It shows that how accurately the SEM can predict the outcomes. The value of R^2^ greater than 0.26 is acceptable (Cohen, 2013, Zhang et al., 2024). Therefore, R^2^ for each hypothesis was greater than 0.665, which confirms the predictive capacity of SEM.

**Table 4:** Direct impact of variables.

Relationships	β	Std. Err.	*t*-value	F^2^	Q^2^	R^2^	Decision
SGI -> SGB	0.454	0.046	9.818	1.784	0.274	0.808	Accepted
GCI -> SGI	0.422	0.104	4.058	1.644	0.352	0.793	Accepted
IES -> SGI	0.273	0.084	3.262	0.571	0.264	0.665	Accepted
ISP -> SGI	0.302	0.094	3.223	1.542	0.304	0.758	Accepted
ISS -> SGI	0.291	0.046	6.326	0.993	0.289	0.746	Accepted

SGB, Students’ green behavior; SGI, Students’ green intentions; IES, Institutional ecosystem; ISP, Institutional sustainability policies; ISS, Institutional support system.

**Figure 2:**
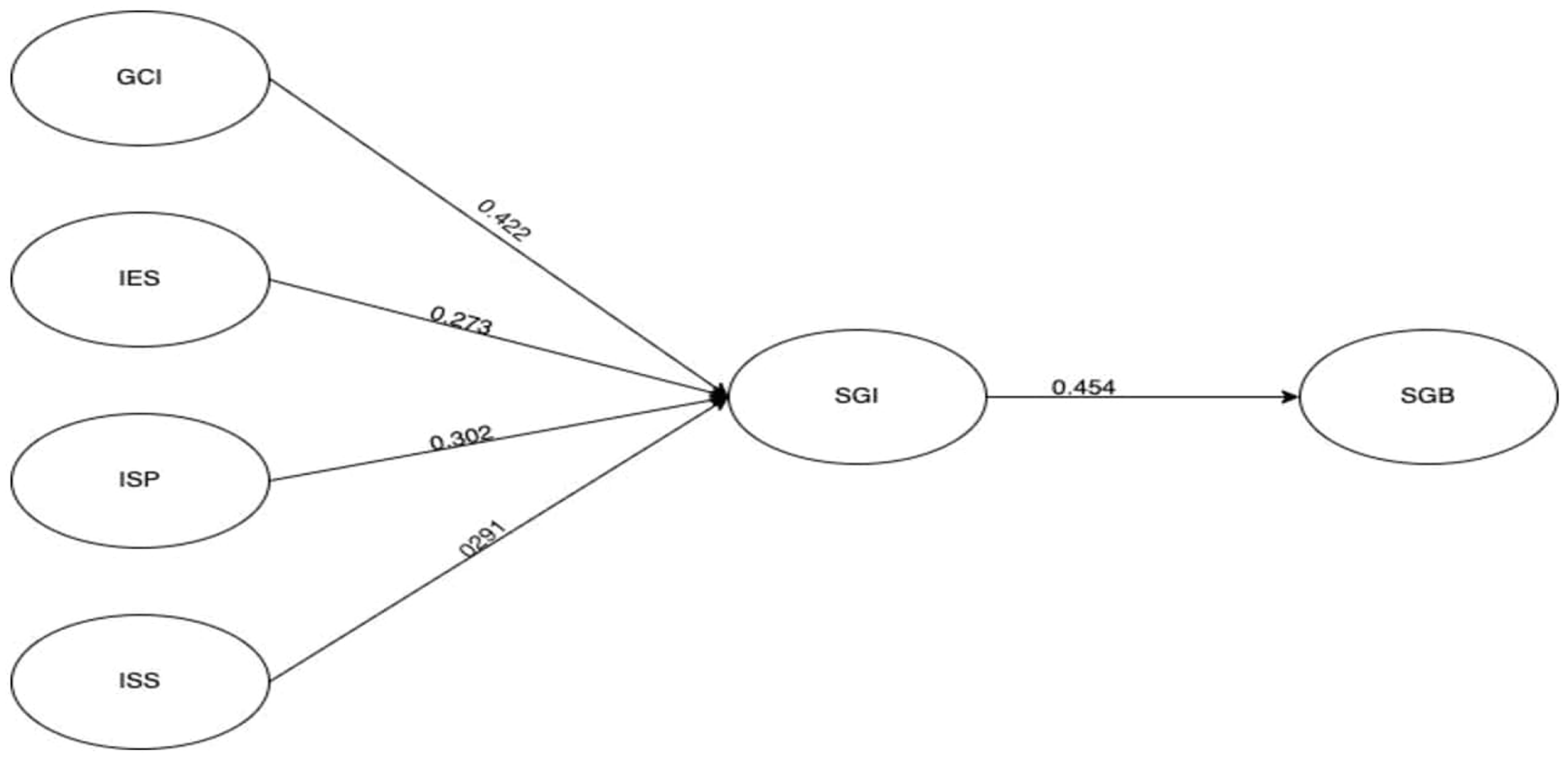
Graphical presentation of PLS-SEM outcomes. SGB, students’ green behavior; SGI, Students’ green intentions; IES, Institutional ecosystem; ISP, Institutional sustainability policies; ISS, Institutional support system.

The findings of SEM reveal that GCI (β = 0.422, t-value = 4.058, *p* < 1%) significantly affects the SGI. It implies that the sustainability-based initiatives at campus greatly matters in developing the students’ green intentions. Similarly, IES (β = 0.273, t-value = 3.262, p < 1%) also have significant positive impact on SGI. This means that the institutional environment must be supportive, which also contributes to the development of students’ green intentions. The significant and positive impact of ISP (β = 0.302, t-value = 3.223, p < 1%) on SGI implies that institutional policies that integrate sustainability with lower environmental implications are important in developing SGIs. Finally, ISS (β = 0.291, t-value = 6.326, p < 1%) also demonstrates the favorable and strong impact of availability and accessibility to resources and infrastructure on the SGI campus. The results for f2 emphasize the effect size of all variables on the SGI. Therefore, F2 indicates that GCI (=1.644), IES (=0.571), ISP (=1.542), and ISS (=0.993) have large effect sizes (>0.35; Cohen, 2013). Similarly, the values of Q2 for all hypotheses are greater than zero (Fornell and Cha, 1994), which confirms the predictive relevance of all constructs. Considering the impact of SGI on SGB, the findings revealed a significant and positive impact of SGI on SGB (β = 0.454, *t*-value = 9.818, *p* < 1%). This implies that the aforementioned factors substantially affect the development of the SGI, which further affects the SGB of students. It also had a large effect size (1.784). Figure 2 provides the graphical presentation of results.

### Moderating effect of EE between SGI and SGB

To analyze the categorical moderation effect of EE between SGI and SGB, the critical ratios for the changes in regression weights between the two EE groups were determined. One group contained students with environmental courses and the other group consisted of students without environmental courses. The determined critical ratios were used to measure the *p*-values, which were further used to estimate the significance of the outcomes. Table 5 indicates that SGI has a positive and significant impact on SGB for both EE groups with environmental subjects (=4.583, *p* < 0.01) and without environmental subjects (=2.38, *p* < 0.01). These findings signify that the effect of SGI on SGB was substantially larger for students whose education included environmental courses than for students whose education did not include environmental courses. Therefore, it confirms that EE is a strong moderator between students’ SGI and SGB.

**Table 5:** Moderating role of environmental education between students’ green intentions and behavior.

Variables	β	Decision
With course	4.583*	Accepted
Without course	2.38*	
Delta *Z* scores	5.49*	

* shows significance level at 1%.

## Discussion

Students are important assets of a country because they represent the next generation of professionals, leaders, and citizens, and they will shape the future of society. With growing concern about climate change around the world, students are expected to play a crucial role in lowering environmental impacts. Therefore, promoting SGB among students not only combats global issues such as climate change but also develops green habits to promote sustainable living. Similarly, developing SGIs is very important because it builds a strong bridge between awareness and actions. It also turns the passive understanding of environmental issues into students’ proactive environmental behavior, leading to a positive change in society. Institutions can play a major role in the SGI leading SGB among students. This study explores how institutions can influence SGI toward developing their SGB. Therefore, this study aimed to analyze the dynamic relationship between GCI, IES, ISP, ISS, SGI, and SGB among students. After confirming the internal consistency and discriminant validity of the effectively measured constructs, the goodness of fit of the structural model was confirmed, allowing us to proceed with further analysis.

The findings reveal a significant positive impact of GCI on SGI, implying that GCI plays a crucial role in the development of SGI. GCI includes green infrastructure that focuses on sustainability, reduces environmental implications, and increases awareness of sustainable development among students (Hayder, 2018; Mafongosi et al., 2018). As students are frequently involved in using recycled bins on campus, they develop their habit of waste management. Moreover, by using the recycling bins directly, students understand the importance of resource conservation, which often extends to their daily routine of their lives beyond campus. Students’ involvement in the initiatives taken by their institutions enhances their awareness and knowledge of their daily routine actions’ environmental impact, which fosters their green intentions and leads to good SGB among students. Thus, universities’ sustainability-based initiatives play a crucial role in enhancing students’ knowledge (Figueredo and Tsarenko, 2013; Andrade, 2021), which significantly contributes to green intentions (Chan et al., 2014).Moreover, the energy saving programs, existence of sustainable infrastructure such as solar panels, water conservation systems, and energy-efficient buildings signify how many educational institutions are committed to environmental sustainability, which motivates their students to understand the importance of the environment (Jung et al., 2019; Al-Naqbi and Alshannag, 2018) and align their actions accordingly. Moreover, when students realize the importance of efforts made by their institutions through green initiatives to lower environmental impact (Ribeiro et al., 2021), they are more likely to develop their habits according to campus-based green practices. Therefore, GCI has a strong impact on the development of the lasting SGI leading to SGB.

The study outcomes indicate a favorable and strong impact of IES on SGI. The IES creates an environment that fosters the sustainable learning of students (Molderez and Fonseca, 2018) and develops a sense of responsibility among students. IES includes the resources, infrastructure, and physical design of the campus, which greatly affect the development of green intentions among students. The social and physical campus environment, along with sustainable infrastructure, shapes students’ behavior. Universities with a sustainable environment promote students’ engagement with environment-oriented activities and enhance their awareness among students (Leal Filho et al., 2018a, 2018b), which directly affects SGI. Green universities that incorporate sustainability into their campus activities, such as infrastructure, research, and facility operations, also greatly contribute to making students more sustainable. Green universities disseminate more information about environmental sustainability, which enhances students’ awareness and knowledge of the sustainable environment (Dagiliūtė et al., 2018).

The significant and positive impact of ISP on SGI can be discussed in terms of its role in developing the environmentally responsible outlook of educational institutions for students. When institutions clearly integrate sustainable policies and strictly implement them on campus, they create a sustainable living environment for students to learn and develop their habits to adopt green practices in their daily actions. For example, universities enforce waste reduction policies and encourage students to participate in waste reduction activities, leading them to take the same action in their routine habits. Therefore, ISP is very important because it offers a basis for systematic initiatives, as institutions with strong ISP are more likely to engage in sustainable practices (Leal Filho et al., 2018a, 2018b), which may force students to act as responsible environmental stakeholders. Vicente-Molina et al. (2018) also demonstrates that educational policies at institutions may inspire the students to engage in sustainable actions.

The findings reveal that ISS has a significant and positive effect on SGI. The higher educational institutions may assist their students by offering the mentorship, resources, and academic short courses that specifically focus on sustainability, and green practices enhancing the knowledge and awareness among students (Mccullough and Pelcher, 2021; Dagiliūtė and Liobikienė, 2015). This institutional support can develop an environmentally conscious mindset that intentionally makes students greener. Similarly, awareness campaigns, seminars, and workshops create environments that teach students the importance of a sustainable environment (Berchin et al., 2018; Radwan and Khalil, 2021). Moreover, when university leaders do not compromise sustainability at campus, they give the environment high priority and inspire the participation of university students, making them more aware of sustainable practices for their daily activities.

The findings also indicate that SGI has a significantly positive impact on SGB. The intentions developed through the awareness and knowledge affected by GCI, IES, ISP, and ISS further lead to consistent actions of the students. This implies that SGI shapes students’ SGB (Lee et al., 2015). Liu et al. (2020) also demonstrates the direct impact of green intentions on green behavior. SGI develops a mindset that makes students more conscious of their routine activities. This SGI further motivates students to adopt green practices that lead to GB. Therefore, when students consistently act on their green intentions, they become rooted in their lifestyles.

The findings support the moderating role of EE between students’ SGI and SGB. The results indicated that students with EE fared better on SGI on SGB than students with no EE. Students may desire to adopt green practices, and they often lack the knowledge and awareness of implementing them effectively. Therefore, EE assists them in understanding environmental issues, finding sustainable and practical solutions, and enabling them to understand the impact of their daily routine (Ma et al., 2023). EE has a substantial impact on skills and knowledge (Kopnina, 2018), which is necessary to act on intentions. Thus, EE empowers students to make informed decisions and bridges the gap between their intentions and actions (Rieckmann, 2018; Reid, 2019). As such, students with EE are equipped with understanding, tools, and confidence to turn their knowledge to their sustainable intentions, leading to meaningful SGB.

Even though the study was conducted with the utmost assistance, it still has some limitations. Research data were collected from students in four Chinese cities with the highest number of HEIs; therefore, the results may have limited general applicability to other cities with diverse institutional cultures and facilities. Moreover, the cross-sectional study limits inference of causality and does not follow changes in SGB over time. Self-reported data may be influenced by social desirability bias, and the study’s focus on institutional factors overlooks other influences such as personal values or societal norms. Additionally, the sample’s homogeneity and emphasis on higher education excludes insights from primary, secondary, or non-formal education contexts. Future research should consider issues in the implementation of green initiatives and cross-country differences in the moderating role of environmental education.

## Conclusion

The development of SGB among students is a crucial factor because students will be the leaders, professionals, and citizens of the next generation. Their institutions are expected to be catalysts of SGI, leading to SGB among the students. The current study considers various factors associated with HEIs that directly affect students’ SGI toward SGB. These factors include GCI, IES, ISP, and ISS. After confirming internal validity and affective measurement of underlying constructs through FL, CR, and AVE, the path coefficients were measured by PLS-SEM. The goodness of fit parameters allowed further analysis.

The findings revealed a significant positive impact of GCI, IES, ISP, and ISS on the SGI. This implies that green initiatives at campus, the green environment at institutions, sustainable policies of universities, and support system availability at universities greatly affect the development of SGI by enhancing students’ awareness and knowledge about sustainable and environmental responsibility as well as green practices. The SGI had a strong direct impact on the SGB of students. ISP demonstrates that students’ green intentions develop an environmentally responsible mindset, which makes them more conscious of their daily actions. Therefore, institutions can play an important role in developing the SGI, and consistently acting on green intentions, SGB becomes embedded in their lifestyle.

The institution has a crucial role in developing the SGI and SGB, and the findings have the following policy implications. Educational institutions must provide facilities to students, such as discounts for reusable materials in cafeterias and fostering energy conservation habits. Moreover, universities can inspire their students to embed green practices in their lives by enhancing their awareness through organizing seminars and workshops. Providing sustainability-led mentorship to students can turn SGI to SGB. Moreover, universities must organize sustainability-focused workshops to teach their students how to adopt green practices consistently into daily routine activities such as energy conservation, growing green gardens, and lowering waste in their living places. Moreover, universities can also provide sustainable food at cafeterias, affordable organic food on campus, and bike-sharing programs, which can develop strong SGI, leading to habitual behavior among students.

## Data Availability

The original contributions presented in the study are included in the article/supplementary material, further inquiries can be directed to the corresponding author.
